# Spontaneous Pneumomediastinum: A Narrative Review Offering a New Perspective on Its Definition and Classification

**DOI:** 10.7759/cureus.81822

**Published:** 2025-04-07

**Authors:** Santiago Campbell-Silva, Sebastián Campbell-Quintero, Diana C Díaz-Rodríguez, Santiago Campbell-Quintero, Iyuleisa Castro-González

**Affiliations:** 1 Internal Medicine, Clínica Mediláser, Florencia, COL; 2 Cardiology, Medilaser Clinic, María Inmaculada Hospital, Florencia, COL; 3 Internal Medicine, Hermanos Ameijeiras Hospital, Havana, CUB

**Keywords:** pneumomediastinum classification, primary pneumomediastinum, secondary pneumomediastinum, spontaneous pneumomediastinum, spontaneous pneumomediastinum definition

## Abstract

Pneumomediastinum is the abnormal presence of air in the mediastinum. It is typically classified into two types: spontaneous (occurring without any underlying lung disease) and secondary (resulting from various conditions, including trauma). The most common clinical symptoms include retrosternal chest pain, difficulty breathing, subcutaneous emphysema in the neck or chest, and, in some cases, Hamman’s sign, which involves crackles that occur in sync with the heartbeat. This study has two main objectives: first, to assess whether there is consistency between the definition of spontaneous pneumomediastinum presented by the authors and the description provided in their articles, and second, to evaluate whether the classification of spontaneous pneumomediastinum aligns with its definition. We conducted a literature search using PubMed, Google Scholar, and the Springer Journal Archive databases.

## Introduction and background

The mediastinum is a compartment of the thoracic cavity, located between the two pleural cavities in a longitudinal orientation. Its lateral boundaries are defined by the pleural cavities, with the upper boundary marked by the thoracic inlet, the posterior boundary by the thoracic spine, the anterior boundary by the sternum, and the inferior boundary by the diaphragm. The mediastinum houses key structures such as the heart, esophagus, trachea, spinal nerves of the thorax, and blood vessels.

The presence of free air within the mediastinum is referred to as pneumomediastinum. The term “pneumomediastinum” was first introduced at the end of the 19th century. At that time, the understanding of pulmonary and thoracic diseases was in its early stages, making the diagnosis of pneumomediastinum quite rare. Despite subsequent scientific advances, however, this condition remains underdiagnosed.

The first description of spontaneous pneumomediastinum is credited to Laennec in 1819 in relation to trauma, where it was referred to as “interlobular emphysema” [1]. However, in 1783, Simmons reported a case of postpartum subcutaneous emphysema, which is likely the first documented case [2]. In 1939, Hammam described seven cases of “spontaneous mediastinal emphysema,” a form of mediastinal emphysema occurring in healthy individuals without any underlying disease. This condition was then referred to as “medical mediastinal emphysema,” a term that excluded traumatic cases [3,4]. Ultimately, the term “medical mediastinal emphysema” was abandoned.

Subsequently, “mediastinal emphysema” was replaced by “pneumomediastinum,” which facilitated the continued use of the term “spontaneous pneumomediastinum.” Although Hamman was not the first to observe the presence of air in the mediastinum, his careful clinical description and distinction of this condition from nontraumatic cases led to the widespread use of the term “spontaneous pneumomediastinum” in the medical literature. In honor of his contributions, “spontaneous pneumomediastinum” has often been referred to as “Hamman’s syndrome.”

In 1944, Macklin and Macklin further advanced the understanding of the pathophysiological mechanism underlying pneumomediastinum, explaining that it results from the rupture of terminal alveoli, causing air to escape into the pulmonary interstitium. This air then migrates along the vascular sheaths from the pulmonary hilum into the mediastinum [5].

The lack of research supporting the current classification, which is based largely on clinical observations, highlights the need for a critical reevaluation of the existing criteria for the definition and classification of pneumomediastinum.

What did we know before this study?

A review of the literature reveals that most articles on pneumomediastinum, particularly those discussing the “spontaneous” form, focus on case studies and series emphasizing clinical manifestations, imaging findings, and treatments. However, these articles fail to thoroughly analyze the true nature of the condition, leading to confusion surrounding the term “spontaneous pneumomediastinum.” This lack of clarity complicates classification and raises conceptual challenges. To address these issues, we conducted an exhaustive review of the literature. Our objective is to propose a novel definition, terminology, and classification system for spontaneous pneumomediastinum, a topic that has remained largely unexplored for over a century.

What is the contribution of this study?

Pneumomediastinum remains a clinically underreported condition. As definitions evolve, it is important that classifications align with accurate definitions. This study presents a fresh perspective on spontaneous pneumomediastinum, aiming to reconcile the misinterpretation of its definition and improve classification based on pathophysiological rather than purely clinical factors. This article not only offers the most comprehensive analysis of spontaneous pneumomediastinum to date but also represents the first major contribution to the specialized literature in this field.

What are the implications of this study?

Although spontaneous pneumomediastinum is rare, this study provides the first update to its definition and offers a clearer understanding of the classification of secondary pneumomediastinum by identifying predisposing and precipitating factors. This contributes to a better understanding and improved communication among healthcare professionals. Further research is needed to explore the etiology of primary pneumomediastinum.

## Review

**Figure 1:**
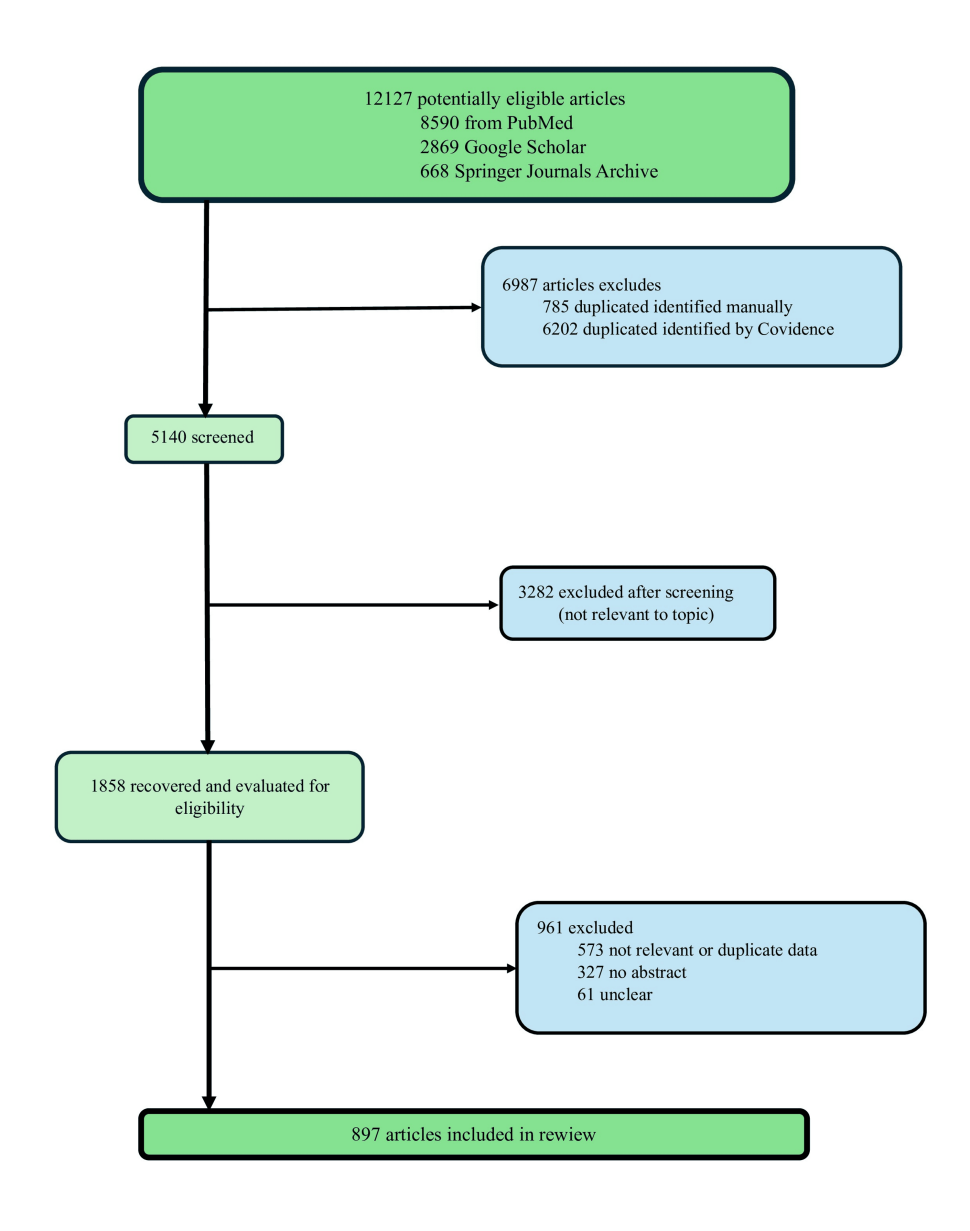
PRISMA flowchart of the study selection process PRISMA, Preferred Reporting Items for Systematic reviews and Meta-Analyses

A total of 12,127 potentially eligible articles were identified, of which 897 were read and analyzed in their entirety. A Preferred Reporting Items for Systematic reviews and Meta-Analyses (PRISMA) flowchart was created to illustrate the study identification and article filtering process, as shown in Figure 1 [6].

**Figure 2:**
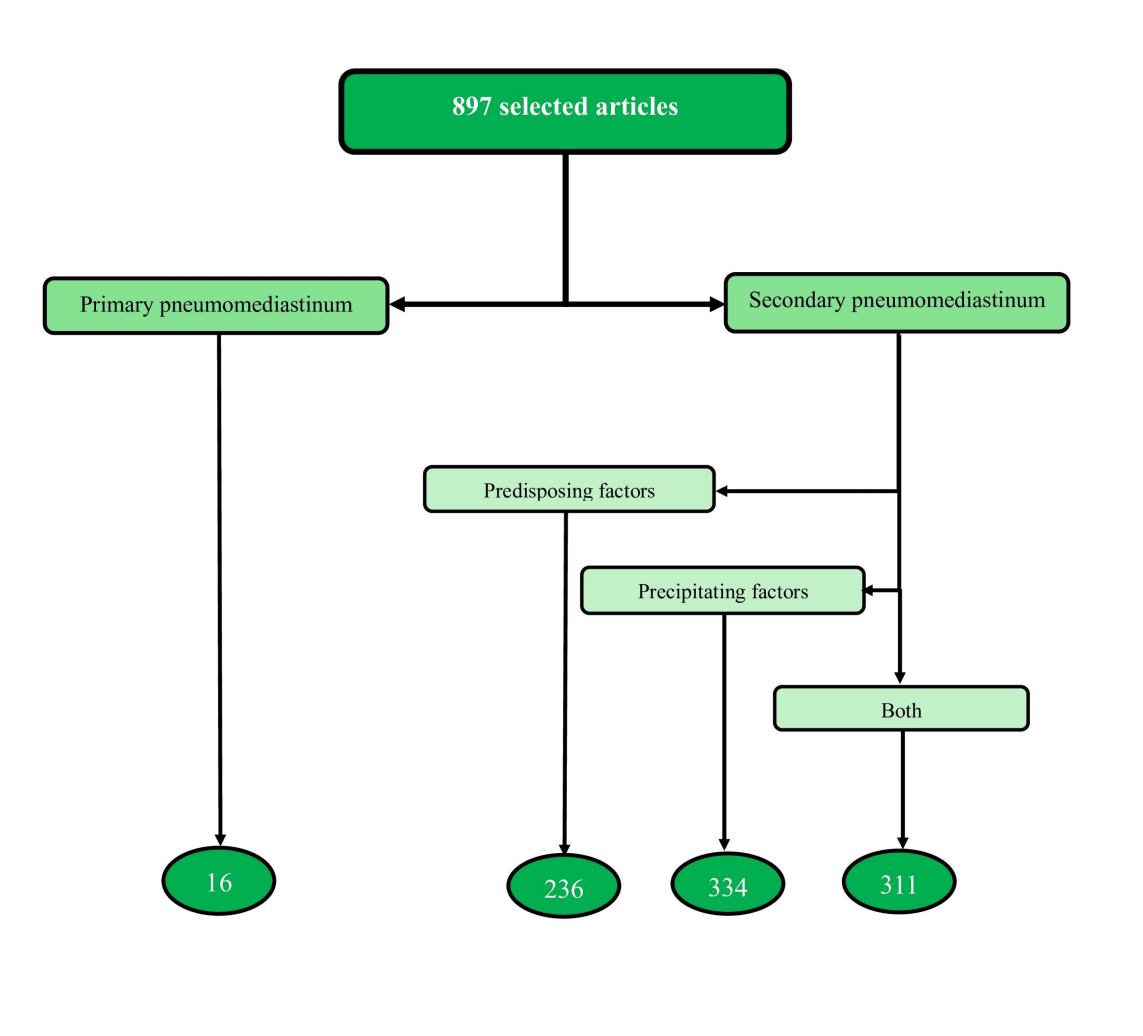
Distribution of articles by type of pneumomediastinum

Out of these 897 articles, only 16 (1.8%) focused on primary (or truly spontaneous) pneumomediastinum. The remaining 881 articles (98.2%) were classified as secondary pneumomediastinum, as shown in Figure 2. A significant difference was observed, with a p-value < 0.05. Table 1 presents additional statistical data from the results.

**Table 1:** Statistical data of selected articles p-Value: <0.05

Articles	Observed frequency	Expected frequency	Frequency ratio (%)	Lower 95% CI (%)	Upper 95% CI (%)	Chi-square
Primary pneumomediastinum	16	16.14	1.78	0.91	2.64	0.0013
Secondary pneumomediastinum (with predisposing factors)	236	236	26.3	23.42	29.19	2.07
Secondary pneumomediastinum (with precipitating factors)	334	334.04	37.23	34.07	40.39	5.48
Secondary pneumomediastinum (with both)	311	310.98	34.67	31.55	37.78	3.28

Discussion

Analysis of the Definition

Definitions and classifications are human interpretations of biological phenomena. While some are effective for specific purposes, others may not be as useful. From a clinical perspective, definitions should aid in providing an accurate diagnosis, treatment, or management. However, medicine often treats standards, protocols, definitions, and classifications as fixed entities, which cannot be changed or modified. These processes must evolve in order to improve our understanding.

Despite the existence of numerous sources addressing aspects of this condition, we did not find articles that specifically analyze the correlation between the current definition of spontaneous pneumomediastinum and what is presented in the published literature. There are no studies that establish a direct link. This gap highlights the importance of conducting this review and updating the existing scientific literature to improve the understanding and characterization of this clinical entity.

We believe the true definition of spontaneous pneumomediastinum comes from Hammam, who described “spontaneous mediastinal emphysema” as mediastinal emphysema occurring in healthy individuals with no demonstrable underlying disease and unrelated to traumatic events [3]. The phrase “without any demonstrable underlying disease” can now be interpreted as the absence of predisposing factors (such as asthma, chronic obstructive pulmonary disease (COPD), etc.).

Many authors [7-10], while acknowledging that this condition occurs without an apparent cause, have associated it with multiple conditions such as asthma, diabetic ketoacidosis, pneumonia, drug inhalation, labor, severe coughing, or vomiting. This weakens the definition of spontaneity, as pneumomediastinum related to an identifiable event cannot be considered truly spontaneous. This demonstrates that there has been and continues to be a misinterpretation of spontaneous pneumomediastinum. The definition has been inaccurately perpetuated, deviating from its original conception. Consequently, this inaccuracy has led to a definition that is both confusing and imprecise. Moreover, the incongruence between the definition and its meaning results in conceptual inconsistencies. Medical language should aim for clarity and precision to avoid confusion and ambiguity [11-13].

However, when pneumomediastinum occurs in healthy individuals without a clearly identifiable cause, why does it happen? What causes the increased intra-alveolar pressure to rupture? Are there subtle or undiagnosed conditions at play? For these rare cases, the underlying abnormal condition would need to be elucidated, which is not an easy task. Until a cause for this type of pneumomediastinum is identified, it should be considered truly spontaneous. The absence of an identifiable causal factor, which is central to the definition of spontaneous pneumomediastinum, presents a diagnostic challenge and an opportunity for further exploration and understanding.

Reports indicate that up to 51% of cases do not have an identifiable cause [14-16], and some of these cases, or even all of them, could correspond to spontaneous pneumomediastinum but were not specified as such. In our study, we found no cause in 16 articles (1.8%), some of which were referenced [17-22]. This type of pneumomediastinum generally does not require surgical intervention unless there is significant cardiorespiratory compromise.

Definition of Primary Pneumomediastinum

The term “spontaneous pneumomediastinum” should be replaced with “primary pneumomediastinum.” This condition should be defined as occurring in healthy individuals without any known predisposing or precipitating factors and without being associated with traumatic events.

Given the low incidence of this event, it is not widely documented in the medical literature, unlike secondary pneumomediastinum, which accounted for 98.2% of cases compared to 1.8% of spontaneous (or, more appropriately, primary) pneumomediastinum in our study. Paradoxically, in the literature, secondary pneumomediastinum is often less documented than spontaneous pneumomediastinum due to its association with traumatic events (whether iatrogenic or non-iatrogenic). In the absence of such trauma, any factor leading to pneumomediastinum (such as predisposing or precipitating factors) is typically classified as spontaneous, which contradicts the definition. Any event associated with the appearance of pneumomediastinum should be considered secondary pneumomediastinum.

Finally, there is a disconnect between the definitions of pneumomediastinum proposed by different authors and how these definitions are expressed in their articles.

Analysis of the Classification

According to our observations, the initial classification of pneumomediastinum as a clinical entity was relatively informal and based on sporadic or anecdotal reports. These reports were later structured into isolated case reports and case series. This trend continues to be observed today, but we found no publications specifically aimed at clarifying the true definition of spontaneous pneumomediastinum or its classification.

The development of classifications has paralleled advancements in diagnostic imaging. Chest radiography, introduced in the 20th century, improved the accuracy of detecting air in the mediastinum. Later, the introduction of computed tomography allowed for more detailed evaluation and confirmation of the diagnosis, even in mild or atypical cases. Moreover, this evolution has been linked to a deeper understanding of the underlying causes and mechanisms of pneumomediastinum.

Early classifications of pneumomediastinum were based on etiological criteria and clinical presentation, establishing two main categories: spontaneous and secondary. As previously noted, spontaneous pneumomediastinum was described as occurring in the absence of a traumatic event and in otherwise healthy individuals, whereas secondary pneumomediastinum was associated with an identifiable cause, typically trauma. The clinical presentation of pneumomediastinum has a broad range of characteristics, which require a high degree of clinical suspicion. While these aspects are still valid today, the initial classification remains inaccurate, which we aim to correct.

We propose that pneumomediastinum, as defined by Hamman as “spontaneous mediastinal emphysema” [3], fits into the first category. Hamman established the foundational distinction between pneumomediastinum with no apparent cause (spontaneous) and that with an identifiable cause (secondary). For nearly a century, pneumomediastinum has been classified into two types: spontaneous and secondary.

Some authors [7,8], like many others, define pneumomediastinum in two categories, but these authors include triggering factors in both groups, which contradicts the definition they provide. A truly spontaneous pneumomediastinum, by its very definition, cannot have any triggering factors. This methodological misstep, which combines predisposing and precipitating factors, can create confusion for readers.

Over time, the classification of secondary pneumomediastinum has become simplified, associating it with traumatic events of any etiology. The spontaneous group is now used to categorize any pneumomediastinum not caused by trauma. Thus, according to current medical literature, any pneumomediastinum that is not traumatic is considered spontaneous. However, this misclassification refers to a group with major triggering causes [11-13].

**Figure 3:**
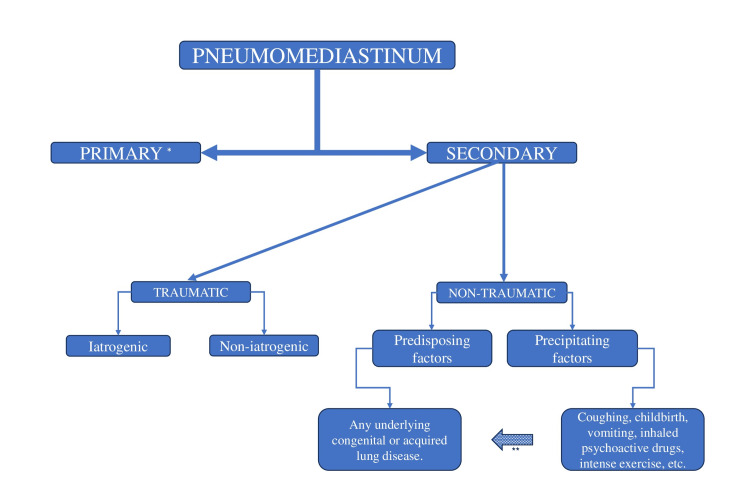
Classification of pneumomediastinum according to the authors * It cannot involve any predisposing factors, precipitating factors, both, or trauma. ** Precipitating factors may act on predisposing factors.

From our observations and the articles we reviewed, we contend that the current “spontaneous pneumomediastinum” is not truly spontaneous but is, in fact, secondary. Therefore, if it is secondary, it cannot also be spontaneous, as these categories are mutually exclusive. Consequently, the classification into “spontaneous” and “secondary” pneumomediastinum is inappropriate and lacks specificity. This classification reflects clinical circumstances rather than pathophysiologic ones. We advocate for a classification system that takes into account not only the etiology of pneumomediastinum but also its pathophysiology. This approach would better guide management strategies, optimize care, and provide a framework that is easier to remember and useful for teaching (Figure 3).

When pneumomediastinum is diagnosed, efforts are made to investigate changes in the integrity of the respiratory or digestive tract, leading to radiological evaluations, dietary restrictions, unnecessary antibiotic administration, and prolonged hospitalizations. However, if we recognize that pneumomediastinum is truly spontaneous, these interventions are unjustified, as the condition typically follows a self-limiting course and, in most cases, does not recur.

If pneumomediastinum is associated with a predisposing factor that compromises pulmonary structure, whether congenital, hereditary, or genetic (such as bronchiectasis, cystic fibrosis, surfactant alterations, etc.) or acquired (such as asthma, COPD, interstitial lung disease, COVID-19 pneumonia, etc.), it cannot be considered spontaneous, as there is an underlying disease. Similarly, pneumomediastinum that occurs due to a precipitating factor (such as coughing, labor, intense exercise, use of inhaled drugs, mechanical ventilation, etc.) in either a healthy individual or one with underlying lung disease cannot be spontaneous, as it is triggered by an immediate event. Likewise, pneumomediastinum associated with other conditions, such as anorexia nervosa, Marfan syndrome, Ehlers-Danlos syndrome, malnutrition, idiopathic inflammatory myopathies, diabetic ketoacidosis, inflammatory bowel disease, and others, cannot be considered spontaneous either (Table 2).

**Table 2:** Some causes of secondary pneumomediastinum Some causes of secondary pneumomediastinum

Predisposing factors	Precipitating factors
Asthma	Coughing
COVID-19	Childbirth
Bronchitis	Choking
Pneumonia	Vomiting
Bronchiectasis	Sneezing
Pulmonary metastases	Intense screaming
Bronchiolitis obliterans	Vigorous crying
Acquired tracheomalacia	Intense exercise
Alpha-1 antitrypsin deficiency	Seizures
Mounier-Kuhn syndrome	Immersion and diving
Idiopathic pulmonary fibrosis	Electronic cigarettes
Pulmonary cystic lesions	Inhalation of toxic gases
Other respiratory infections	Inhaled psychoactive drugs
Interstitial lung disease	Vigorous sports (weightlifting)
Primary pulmonary malignancies	Forced defecation
Fibrosis due to irradiation or other causes	Other Valsalva maneuvers
COPD	Inhalation of helium gas (party balloons)

There are authors who refer to spontaneous pneumomediastinum as “idiopathic spontaneous” [19] or “primary spontaneous” [23,24]; however, these terms are redundant. The word “spontaneous” already implies that something occurs without a known cause, “primary” indicates the absence of an underlying disease, and “idiopathic” denotes a condition with no determined cause or origin. Therefore, the terms “primary spontaneous” or “idiopathic spontaneous” are pleonasms. Unfortunately, this is not the only instance of such redundant terminologies being used to describe the same condition, further contributing to the confusion (Table 3).

**Table 3:** Terms used for spontaneous pneumomediastinum

Synonym
Idiopathic spontaneous pneumomediastinum
Secondary spontaneous pneumomediastinum
Acute spontaneous mediastinal emphysema
Primary spontaneous pneumomediastinum
Spontaneous mediastinal emphysema
Spontaneous pneumomediastinum
Medical mediastinal emphysema
Malignant interstitial emphysema
Idiopathic pneumomediastinum
Primary pneumomediastinum
Mediastinal emphysema
Hamman’s syndrome

Pneumomediastinum Must Be Reclassified

We strongly recommend using the term “primary pneumomediastinum” instead of “spontaneous pneumomediastinum” for cases that are truly spontaneous. The term "primary" encompasses what is commonly referred to as spontaneous, idiopathic, mediastinal emphysema, or Hamman’s syndrome. We prefer “primary” as a unified term to avoid confusion and to prevent the use of multiple names or eponyms for the same condition.

A critical distinction must be made regarding the statement that spontaneous (primary) and secondary pneumomediastinum are subject to different etiologies [23,25]. Pneumomediastinum with multiple etiologies is always secondary, never primary. In our study, the leading cause of secondary pneumomediastinum was precipitating factors, which accounted for 334 (37.2%) cases (Figure 2). This distinction is crucial for guiding treatment: for primary pneumomediastinum, the treatment approach is generally straightforward, whereas, for secondary pneumomediastinum, treatment depends on the underlying cause. This confusion arises from the incorrect application of the primary pneumomediastinum definition and, more broadly, from the misclassification of pneumomediastinum.

Despite significant advances in patient care and treatment, the definition and classification of pneumomediastinum have remained unchanged, and there has been little progress toward greater precision.

We believe that medical disorders should be named in a way that accurately reflects their underlying pathophysiology. When traditional names create confusion between different pathophysiologic processes, renaming the condition is preferable.

Finally, the classification of pneumomediastinum is not aligned with the definitions provided by different authors.

## Conclusions

Based on the data obtained and analyzed in this study, it is clear that the current definition of pneumomediastinum is misinterpreted, and the existing classification is inadequate due to a misunderstanding of the definition. The inclusion of triggering factors in the diagnosis of “spontaneous pneumomediastinum” has significantly contributed to the complexity of its understanding, making the condition even more heterogeneous. Therefore, it is crucial to establish a classification system that is simple, easy to remember, and grounded in the correct application of the definition.

Given the evident diversity of opinions among different authors on this subject, it is essential to reach a solid and well-supported consensus in order to effectively and satisfactorily resolve these discrepancies.
